# Efficacy and safety of Brivaracetam as adjunctive therapy in pediatric epilepsy: A systematic review and meta-analysis

**DOI:** 10.1007/s10072-025-08185-9

**Published:** 2025-05-03

**Authors:** Malak A. Hassan, Abdelaziz A. Awad, Ahmed Marey, Ahmed Mostafa Amin, Ahmed Elshahat, Mohamed El-Moslemani, Ahmed Mansour, Shrouk Ramadan, Mohamed A. Aldemerdash

**Affiliations:** 1 Faculty of Medicine, Alexandria University, Alexandria, Egypt; 2 Faculty of Medicine, Al-Azhar University, Cairo, Egypt; 3 Faculty of Medicine, Al-Azhar University, Damietta, Egypt; 4 Faculty of Medicine, Ain Shams University, Cairo, Egypt; 5 Faculty of Medicine, Sohag University, Cairo, Egypt

**Keywords:** Epilepsy, Brivaracetam, Levetiracetam, Systematic review

## Abstract

**Background:**

Brivaracetam is a novel third-generation antiseizure medication and an analog of levetiracetam with selective affinity for synaptic vesicle protein 2A (SV2A). By binding SV2A, brivaracetam decreases pre-synaptic neurotransmitter release.

**Aim:**

We aimed to assess the safety and efficacy of brivaracetam in pediatric epilepsy.

**Method:**

We searched PubMed, Scopus, and Web of Science (WOS) for relevant clinical and observational studies from inception until February 2024. We carried out statistical analysis using Open Meta-Analyst. Dichotomous data were pooled as proportions with a 95% confidence interval (CI).

**Results:**

Eleven studies with a total of 805 patients were identified. The analysis of four studies revealed the more than 50% responder rate in a cohort of 252 focal epilepsy patients to be 51.5% (95% CI: [32.6%, 70.5%]). The analysis of three studies involving a cohort of 266 patients found a 20.7% incidence (95% CI: [15.8%, 25.6%]) of complete seizure freedom. The analysis of nine studies involving a cohort of 737 epilepsy patients revealed a retention rate of 66% (95% CI: [40%, 92%]).

**Conclusion:**

This study highlights the efficacy, tolerability, and safety of brivaracetam as adjunctive therapy in pediatric patients with epilepsy. The findings support its consideration as a valuable treatment option for children and adolescents, particularly those with drug-resistant epilepsy. Further trials with longer follow-up durations are needed to study the optimal doses and explore factors affecting drug response.

**Supplementary Information:**

The online version contains supplementary material available at 10.1007/s10072-025-08185-9.

## Introduction

Affecting more than 50 million people around the world, epilepsy is among the most prevalent neurological disorders [1]. Recent estimates indicate that more than 470,000 children in the United States alone have active epilepsy [2]. In addition to the high incidence of epilepsy in early childhood, more than a third of affected children under the age of three develop drug-resistant epilepsy [3]. Thus placing them at a greater risk for impaired cognitive function and premature mortality [3]. Epileptic syndromes with onset during childhood are of varying etiologies, which may be genetic, structural, metabolic, immune, infectious, and unknown etiologies [4]. The latest classification outlined by the International League Against Epilepsy (ILAE) categorizes pediatric epilepsy syndromes into self-limited focal epilepsies, genetic generalized epilepsy syndromes, and epileptic encephalopathies [4]. Among the most prominent epileptic encephalopathies are Lennox–Gastaut syndrome (LGS), developmental epileptic encephalopathy with spike-and-wave activation in sleep (DEE-SWAS), epileptic encephalopathy with spike-and-wave activation in sleep (EE-SWAS), epilepsy with myoclonic, atonic seizures (EMAtS), hemiconvulsion–hemiplegia–epilepsy syndrome (HHE), and febrile infection-related epilepsy syndrome (FIRES) [4].

The disruption of the balance between excitatory (glutamate) and inhibitory (γ-aminobutyric acid) neurotransmitters is believed to play a role in the pathogenesis of epilepsy [5]. Thus, many antiseizure medications (ASMs) aim to modulate this interaction by targeting voltage-gated channels, GABA receptors, glutamate receptors, or other molecular targets [5].

Brivaracetam, a novel third-generation ASM and an analog of levetiracetam, presents a glimpse of hope for children with epilepsy [6]. Though its mechanism of action remains unclear, its selective affinity for synaptic vesicle protein 2 A (SV2 A) is likely responsible for the observed antiseizure effects [6]. SV2 A stimulates vesicles'maturation and regulates neurotransmitter release [7]. By binding SV2 A, brivaracetam decreases pre-synaptic neurotransmitter release [7]. Brivaracetam has advantageous pharmacokinetic properties featuring rapid absorption and nearly perfect bioavailability [8]. Recently, the oral formulation of brivaracetam has been approved by the Food and Drug Administration (FDA) as monotherapy or adjunctive therapy in the treatment of partial-onset seizures in patients above the age of four [9]. The injection form of brivaracetam remains indicated only in patients above the age of 16 due to the lack of sufficient evidence on its safety in the pediatric population [9].

Though brivaracetam appears to be promising in pediatric epilepsy, it is challenging to derive meaningful conclusions from individual studies. This systematic review and meta-analysis aimed to assess the safety and efficacy of brivaracetam in pediatric epilepsy. In doing so, we can consolidate existing evidence and highlight areas for future research.

## Methods

This systematic review and meta-analysis adhered to the guidelines of the Preferred Reporting Items for Systematic Reviews and Meta-Analyses (PRISMA) statement [10]. The Cochrane Handbook for Systematic Reviews of Interventions served as the primary reference for all steps of this study [11]. This study was registered with PROSPERO ID CRD42024570703.

### Information sources and search strategy

We performed a comprehensive literature search of PubMed, Scopus, and Web Of Science (WOS) from inception till February 2024 using the following strategy: (Brivaracetam OR"UCB 34714"OR broviac OR Nubriveo OR Brivajoy OR breslera OR brisee OR brevetoxin) AND (Pediatri* OR child OR children OR child*) AND (Epilep* OR seizur*).

### Eligibility criteria

Clinical trials and observational studies meeting our PICO criteria were included in this meta-analysis. Population (P): Pediatric patients with epilepsy aged < 18 years; Intervention (I): Brivaracetam; Comparator(C): Any comparator or no comparator; Outcomes (O): Safety and efficacy outcomes, including responder rate, seizure freedom rate, retention rate, side effects, treatment-emergent adverse events (TEAEs), and trial withdrawal due to poor efficacy or TEAEs. Conference abstracts, case reports, animal studies, non-English studies, non-peer-reviewed studies, and studies involving patients over 18 years were excluded.

### Study selection and data extraction

Duplicate records were removed using Endnote software (Clarivate Analytics, Philadelphia, USA). The retrieved records underwent a two-stage screening process. Initially, the studies underwent title and abstract screening by two independent authors to ascertain their relevance [AMA and AE]. In the second stage, the full-text articles were screened to confirm their eligibility for inclusion in our analysis. Any conflicts were resolved through discussion and consensus, consulting a third author [AA] when needed. The screening process was carried out using Rayyan software [12]. A pre-designed Excel sheet was utilized to extract relevant data: a summary of included studies, baseline patient characteristics, and outcomes of interest.

### Outcome definitions

Encephalopathy was defined as a group of disorders of the CNS in which the epileptic activity contributes to severe cognitive and behavioral impairments to a greater extent than what would be expected from the underlying etiology alone.

Combined Generalized and Focal Epilepsies were defined as patients with both generalized and focal seizures. The diagnosis is made on clinical grounds and supported by EEG findings.

### Risk of bias and quality assessment

Two blinded investigators assessed the quality of the included studies [MAH and SR], and a third investigator [AAA] was consulted to resolve discrepancies when needed. We used the Cochrane ROBINS‐I tool to evaluate the risk of bias in nonrandomized clinical trials [13]. This tool assesses bias arising from several domains: confounding factors, selection of participants, defining the interventions, deviations from planned interventions, incomplete data, outcome measurement, and selective reporting. Observational studies were assessed using the Newcastle–Ottawa Scale (NOS) tool [14]. This tool uses a star-based system to determine the following domains: the selection process, the comparability of the study groups, and the ascertainment of the exposure in case–control studies or the ascertainment of the outcome in cohort studies.

### Statistical analysis

Our statistical analysis employed Open Meta-Analyst [15]. Dichotomous data were pooled as proportions with a confidence interval (CI) of 95%. To assess heterogeneity, we utilized the Chi-square and I-square tests. Heterogeneity was considered significant if *P* < 0.1. The fixed-effects model was utilized when data was homogenous. Data with significant heterogeneity were analyzed using the random effects model. We employed a leave-one-out sensitivity analysis to address heterogeneity, excluding one study from the pooled analysis.

## Results

### Literature search

**Fig. 1:**
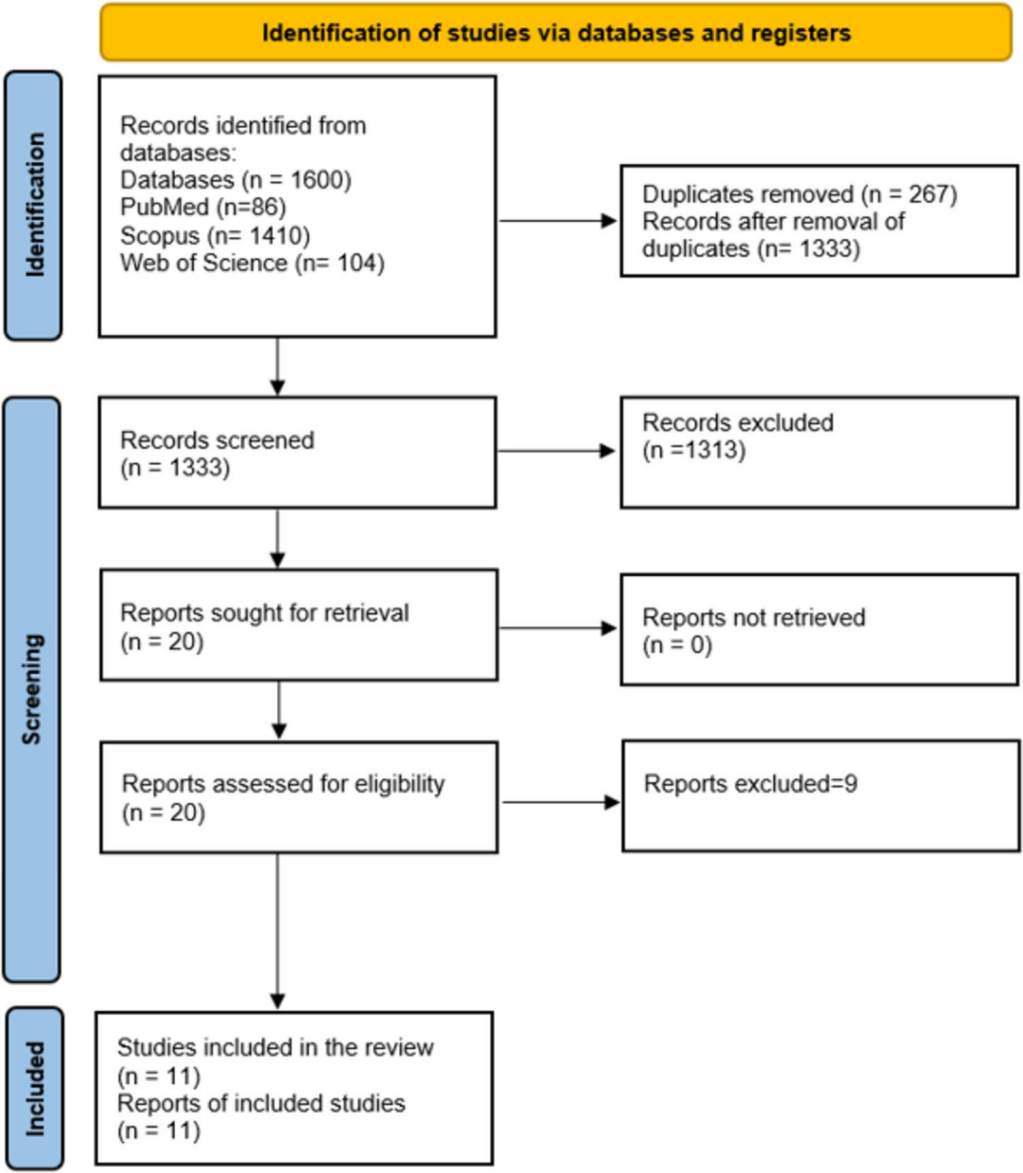
The PRISMA flow diagram demonstrating the search and screening process

Our search identified 390 articles, out of which 136 were duplicates. After screening the titles and abstracts of 254 articles, 18 studies were selected for full-text screening. Finally, we included 11 studies [16–26] in our systematic review and meta-analysis (Fig. 1).

### Baseline characteristics

The included studies involved a total of 805 patients with epilepsy. The mean age of the cohort was 14.4 years. Of the 805 patients, 425 (52.79%) were males. The included patients had various etiologies and epilepsy durations. Five studies [19–21, 23, 24] reported the proportion of drug-resistant patients with (93.4%, 100%,100%, 100%, and 74%) respectively. Four studies [16, 18, 20, 24] reported that 51.5%, 50.2%, 55%, and 18% of patients used valproate. They also reported 17.2%, 23.7%, 30%, and 35%. Three studies reported that 27.3%, 24.1%, and 24.1% utilized topiramate. Regarding doses, Farkas [17] used 4 or 5 mg/kg/day, which is most similar to McGuire [23], Ferretjans [21], and Lagae [16] Ferretjans 3.9, 4.3, and 5 mg/kg/day, respectively. Russo [19] used 1–2 mg/kg/day and increased up to 4 mg/kg/day based on clinical response, While Caraballo [20] used 1.8–10 mg/kg/day. Finally, Liu [18] used 0.8, 1.6, and 3.2 mg/kg/day for patients aged 8 years or more and 1.0, 2.0, and 4.0 mg/kg/day for patients younger than 8. More baseline characteristics are highlighted in Table 1.

**Table 1:** Characteristics of the included studies [16–26]

Study ID	Study design	Country	Sample size	Age, mean (SD) [range], years	Sex, Male, n (%)	Weight, mean (SD), kg	Epilepsy duration, mean (SD), years	Age at diagnosis, mean (SD), years	Epileptic etiology (n%)				
									Focal	Generalized	Encephalopathy	Unclassified	
Farkas 2021	Phase 2 open-label trial	Seven countries (Czech Republic, Germany, Hungary, Italy, Mexico, Spain, and the United States)	50	8.3 (4.0)	26 (52)	NA	4.3 (3.8)	2.6 (3.4)	50 (100)	0 (0)	0 (0)	0 (0)	
Liu 2018	Phase 2 open-label trial	USA, Mexico, Belgium, Czech Republic, Poland, and Spain	99	6.3 (4.8)	49 (48.5)	24.2 (16.2)	3.8 (3.7)	2.5 (3.3)	66 (66.7)	47 (47.5)	0 (0)	6 (6.1)	
Ferretjans 2021 [21]	Retrospective and Descriptive Study	Spain	66	8.65 (3.2)	43 (65)	NA	7.2 (3.4)	2.5 (0.6)	26 (39.3)	13 (19.7)	27 (40.9)	NA	
Lagae 2023 [16]	Phase 3 open-label trial	46 sites in North America, Latin	257	8 (4.5)	141 (54.9)	30.4 (20.2)	4.8 (3.8)	3.3 (3.3)	185 (72)	68 (26.5)	4 (1.5)	0 (0)	
		America, and Europe											
Caraballo 2024 [20]	Retrospective analysis	Argentina	42	9 (3.2)	26 (61.9)	NA	5.2 (2.9)	5 (4.5)	0 (0)	0 (0)	42 (100)	0 (0)	
Hirsch 2018 [22]	Retrospective analysis	Germany	102	42.5 (15.8)	49 (48)	NA	17.6 (15.4)	NA	83 (81.4)	9 (8.8)	0 (0)	8 (7.8)	
McGuire 2020 [23]	Retrospective analysis	USA	20	12.5 [ 4-20]	7(35)	NA	NA	NA	11(55)	6(30)	NA	3(15)	
Russo 2022 [19]	retrospective observational	Italy	45	12.4(4.4)	28(62)	NA	NA	NA	14(31)	2(4.4)	29(64)	NA	
Schubert-Bast 2018 [24]	retrospective observational	Germany	34	12.2(4.2)	14(44.1)	NA	5.2(4.1)	7.1(4.5)	34(100)	NA	NA	NA	

Study ID	Number of previous AEDs used, n (%)				Number of concomitant AEDs, n (%)			Dose (mg/kg/day), mean (SD)	Follow-up (Weeks)	Route of administration	Duration of treatment	Proportion of drug-resistant patients	Other drugs patients were on
	1	3-Feb	5-Apr	> 6	1	2	3 or more						
Farkas 2021	NA	NA	NA	NA	49 (98.0)	NA	NA	4 or 5 mg	2	15-min infusion OR bolus	4.0 (3.2) days	NA	Previous or concomitant, *n* (%) = 49 (98.0)
Liu 2018	37 (37.4)	32 (32.3)	0 (0)	30 (30.3)	32 (32.3)	41 (41.4)	26 (26.3)	0.8, 1.6, and 3.2 mg/kg/day for patients aged ≥ 8 years, and 1.0, 2.0, and 4.0 mg/kg/day for patients aged < 8 years	3	oral solution	18.5 (5.6) days	NA	Valproate = 51 (51.5), Topiramate = 27 (27.3), Lamotrigine = 17 (17.2), Clobazam = 14 (14.1), Phenobarbital = 14 (14.1), Oxcarbazepine = 13 (13.1)
Ferretjans 2021 [21]	4 (6.1)	14 (21.2)	0 (0)	48 (72.7)	13 (20)	27 (40)	23 (34.5)	5.3 (2.2)	15.9 (3.6)	NA	NA	62 (93.4%)	No drugs = 5.5%, 1 drug = 20%, 2 drugs = 40%, 3 drugs = 25%, 4 or more = 9.5%
Lagae 2023 [16]	76 (29.6)	115 (44.7)	0 (0)	66 (25.7)	255 (99.2)	NA	NA	5	9.5 years	tablets or oral solution	NA	NA	Valproate = 129 (50.2), Clobazam = 72 (28.0), Diazepam = 68 (26.5), Topiramate = 62 (24.1), Lamotrigine = 61 (23.7), Phenytoin = 56 (21.8), Carbamazepine = 53 (20.6), Oxcarbazepine = 50 (19.5), Lacosamide = 38 (14.8), Clonazepam = 29 (11.3), Phenobarbital = 26 (10.1), Vigabatrin = 26 (10.1)
Caraballo 2024 [20]	NA	NA	NA	NA	NA	NA	NA	6.7 (2.1)	84	NA	19.5 (5.5) months	42(100%)	Valproic acid in 55%, Clobazam in 40%, Topiramate in 33%, Lamotrigine in 30%, Sulthiame in 20%, Zonisamide in 10%, Cannabidiol in 10%, Perampanel in 5%, Ethosuximide in 5%
Hirsch 2018 [22]	NA	NA	NA	NA	NA	NA	NA	NA	12	NA	301.6 (156.8) Days	NA	Number of concomitant AEDs M (SD) = 1.6 (1.9)
McGuire 2020 [23]	NA	NA	NA	NA	NA	20(100)	NA	3.9(2.3)	NA	NA	8.2 months	20(100%)	range 0–6 antiepileptic drugs but not specified
Russo 2022 [19]	NA	NA	NA	NA	NA	NA	NA	2	24	NA	1 month	45(100%)	NA
Schubert-Bast 2018 [24]	15(53)	13(47)	NA	NA	NA	NA	NA	NA	12	NA	180 days	25 (74%)	LEV = 20 (59) lamotrigine = 12 (35), lacosamide = 9 (27), oxcarbazepine = 8 (24), valproate = 6 (18), carbamazepine = 4 (12)

### Risk of bias assessment

Three studies [16–18] were evaluated using the ROBINS-I tool and showed an overall low risk of bias (Supplementary Table 1S). Eight studies [19–26] were assessed using the NOS tool and demonstrated good quality (Supplementary Table 2S).

### Meta-analysis

#### Responder rate more than 50%

##### With regards to focal epilepsy

**Fig. 2:**
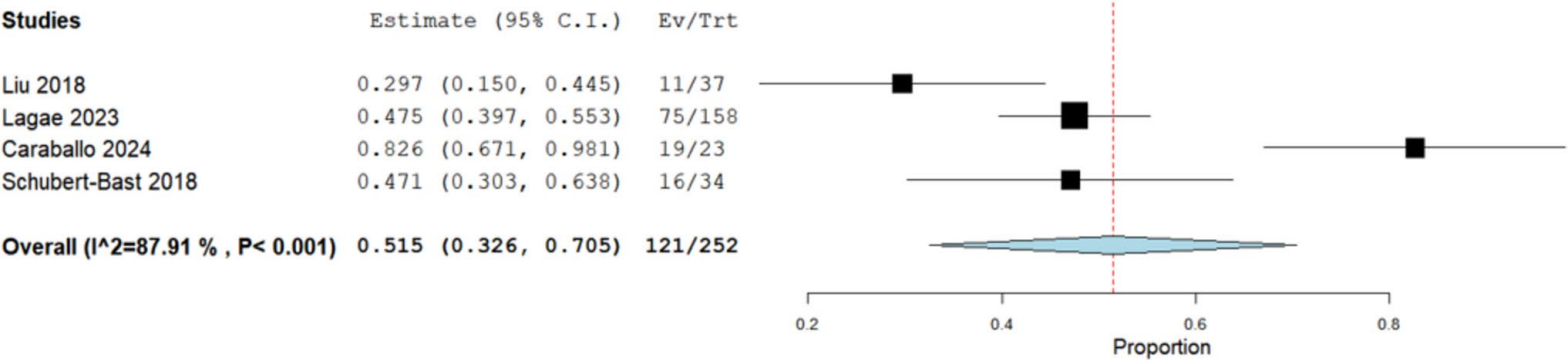
Analysis of the more than 50% responder rate in focal epilepsy

Our analysis of four studies involving a cohort of 252 patients revealed the incidence of more than 50% responder rate to be 51.5% (95% CI: [32.6%, 70.5%]) (Fig. 2). Data was heterogeneous (I^2 = 87.91%, *P* < 0.001). The heterogeneity was resolved by removing Caraballo 2024 (12 months) with a 42.2% incidence (95% CI: [31.2%, 53.3%]) of more than 50% responder rate (I^2 = 55.51%, *P* = 0.106) (Fig. 1S).

##### Regarding generalized epilepsy

One study by Hirsch et al. (2018) reported a more than 50% responder rate in eight patients out of 12. At the same time, a lower than 50% responder rate occurred in four patients out of 12.

##### Regarding encephalopathy

One study reported a more than 50% responder rate in four patients out of nine, while a more than 25% responder rate occurred in one out of nine patients.

##### With regards to combined types of epilepsy

**Fig. 3:**
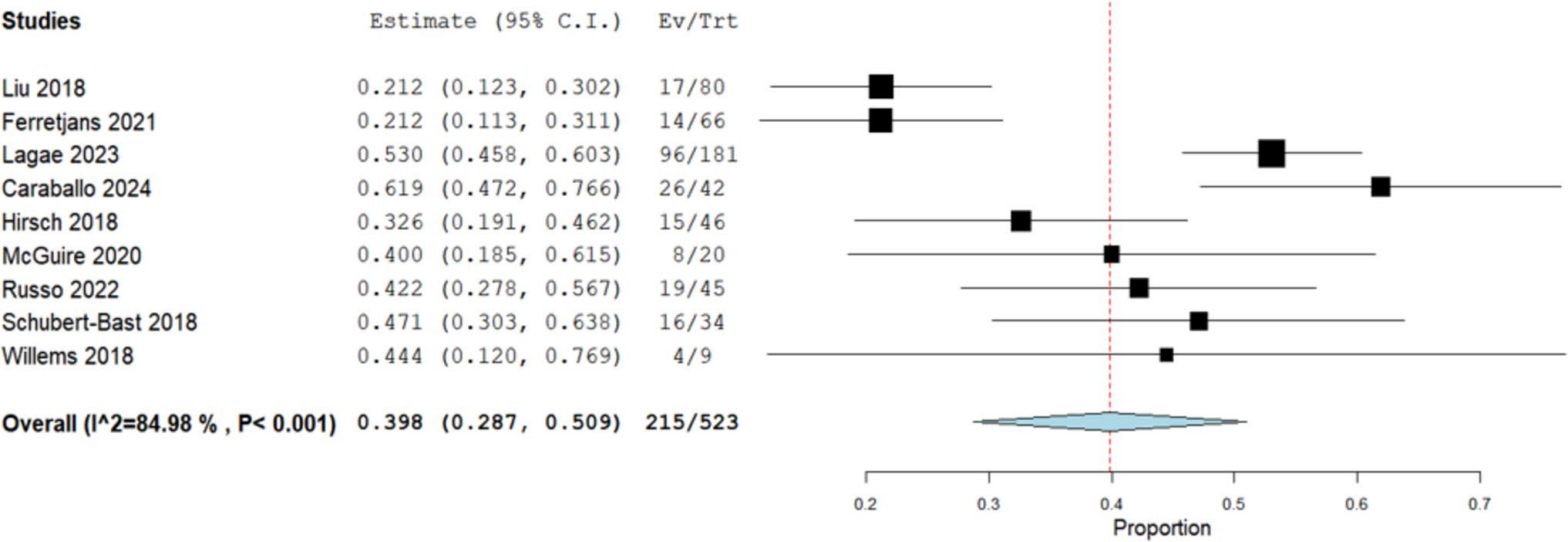
Analysis of complete seizure freedom in focal epilepsy

The analysis of nine studies involving a cohort of 523 epilepsy patients revealed the incidence of more than 50% responder rate to be 39.8% (95% CI: [28.7%, 50.9%]) (Fig. 3). Data was heterogeneous (I^2 = 84.98%, *P* < 0.001). After conducting a leave-one-out analysis, the heterogeneity was attributed to three studies (Liu 2018, Ferretjanas 2021, and Caraballo 2024). Once the heterogeneity was resolved (I^2 = 36.19%, *P* = 0.165), the incidence of more than 50% responder rate was 44.6% (95% CI: [37%, 52.2%]) (Fig. 2S).

#### Complete seizure freedom

##### With regards to focal epilepsy

**Fig. 4:**
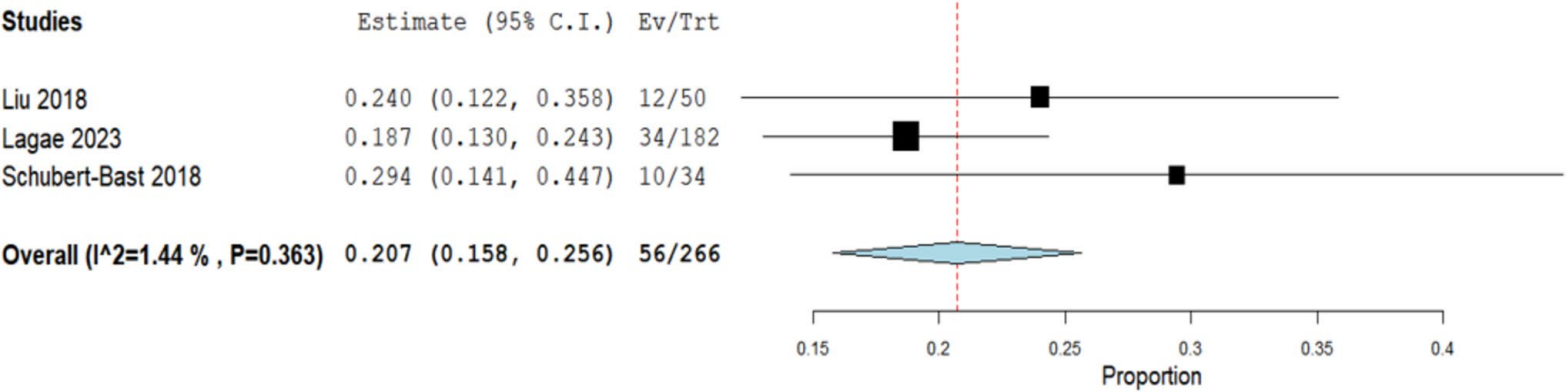
Analysis of the more than 50% responder rate in combined epilepsy types

The analysis of three studies involving a cohort of 266 patients found the incidence of complete seizure freedom to be 20.7% (95% CI: [15.8%, 25.6%]) (Fig. 4).

##### With regards to combined epilepsy types

The analysis of nine studies with a cohort of 647 epilepsy patients revealed an 11.7% incidence (95% CI: [7.6%, 15.8%]) of complete seizure freedom (Fig. 3S). Data was heterogeneous (I^2 = 70%, *P* < 0.001). According to our leave-one-out analysis, Schubert-Bast 2018 and Hirsch 2018 were the sources of the heterogeneity. After resolving the heterogeneity (I^2 = 39%, *P* = 0.104), the incidence of complete seizure freedom was found to be 10% (95% CI: [7%, 13%]) (Fig. 4S).

#### Trial withdrawal due to TEAEs

The analysis of nine studies involving a cohort of 679 epilepsy patients revealed a 10% incidence (95% CI: [5%, 15%]) of trial withdrawal due to TEAEs among those treated with brivaracetam (Fig. 5S). Data was heterogeneous (I^2 = 83%, *P* < 0.001). After conducting a leave-one-out analysis, Farkas 2021, McGuire 2020, and Visa-Rene 2020 were the source of the heterogeneity. After resolving the heterogeneity (I^2 = 15%, *P* = 0.31), the incidence of trial withdrawal due to TEAEs was still 10% (95% CI: [7%, 13%]) (Fig. 6S).

#### Trial withdrawal due to poor efficacy

The analysis of 10 studies involving a cohort of 789 epilepsy patients revealed a 14% incidence (95% CI: [8%, 20%]) of trial withdrawal due to lack of efficacy (Fig. 7S). Data was heterogeneous (I^2 = 90%, *P* < 0.001). After conducting a leave-one-out analysis, the heterogeneity was attributed to three studies (Farkas 2021, Liu 2018, Hirsch 2018). After resolving the heterogeneity (I^2 = 34%, *P* = 0.16), the incidence of trial withdrawal due to poor efficacy was 20% (95% CI: [15%, 25%]) (Fig. 8S).

#### Retention rate

The analysis of nine studies involving a cohort of 737 epilepsy patients revealed a 66% (95% CI: [40%, 92%]) retention rate (Fig. 9S). Data was heterogeneous (I^2 = 99%, *P* < 0.001). The heterogeneity was resolved by removing six studies (Farkas 2021, Liu 2018, Lagae 2023, Russo 2022, Schubert-Bast 2018, and Visa-Reñé 2020). After resolving the heterogeneity (I^2 = 2%, *P* = 0.36), the retention rate was 70% (95% CI: [64%, 77%]) (Fig. 10S).

### Safety outcomes

We summarized the adverse events reported in the included studies (Table 2). In a cohort of 557 patients from eight studies, mental/behavior disorders occurred in 38 patients (6.8%). Somnolence was reported in 44 out of a total of 544 patients (8.09%) across six studies.

**Table 2:** Summary of adverse events

Adverse event	Studies reported	Incidence rate in the Brivaracetam group (Event/Total, (%))
*Mental/behavior disorders rate*	*8*	38/557, (6.8%)
*Somnolence rate*	*6*	44/544 (8.09%)
*TEAEs*	*5*	331/484 (68.39%)
*Serious TEAEs*	*3*	92/406 (22.66%)
*Severe TEAEs*	*3*	48/406 (11.82%)
*Drug-related TEAEs*	*3*	121/406 (29.8%)
*Drug-related serious TEAEs*	*2*	6/356 (1.69%)
*TEAEs leading to discontinuation*	*3*	37/406 (9.11%)

TEAEs were observed in 331 out of 484 patients (68.4%) from five studies. Serious TEAEs affected 92 out of 406 patients (22.7%) from three studies. Among the same 406-patient cohort, severe TEAEs were noted in 48 patients (11.8%). More specifically, drug-related TEAEs were experienced by 121 out of 406 patients (29.8%), and among them, 6 out of 356 patients (1.7%) experienced serious drug-related TEAEs. Three studies reported TEAEs leading to discontinuation in 37 out of 406 patients (9.11%).

## Discussion

Our meta-analysis aimed to assess the safety and efficacy of brivaracetam as adjunctive therapy in pediatric epilepsy. Upon analyzing data from 11 studies involving a total of 805 patients with epilepsy, several key findings emerged.

A responder rate of more than 50% was observed in 51.5% of children with focal epilepsy and 39.8% of children with combined types of epilepsy taking Brivaracetam as adjunctive therapy. To put this into perspective, a meta-analysis on Levetiracetam as adjunctive treatment in children with focal onset seizures reported a 50% responder rate in 56% of patients, suggesting a similar efficacy [27]. Our meta-analysis on Brivaracetam as adjunctive therapy in pediatric epilepsy found that complete seizure freedom was achieved in 20.7% of children with focal epilepsy and 11.7% of children with combined types of epilepsy. In comparison, complete seizure freedom in pediatric epilepsy patients on Lacosamide was reported to be at 14% [28]. These findings suggest that brivaracetam can be an effective treatment option for children with different types of epilepsy. Several factors may have contributed to the observed outcomes.

Firstly, the varying responder rates observed across different types of epilepsy underscore the heterogeneous nature of the disorder and the complexity of treatment outcomes. Focal epilepsy, generalized epilepsy, and encephalopathy may exhibit distinct pathophysiological mechanisms and clinical presentations, influencing individual responses to adjunctive therapy with brivaracetam [29]. The differences in underlying etiology, seizure types, and patient demographics among the included studies may further contribute to the observed variability in treatment response. This emphasizes the need for individualized approaches in managing pediatric epilepsy.

Moreover, the effectiveness of brivaracetam in achieving seizure control and complete seizure freedom in focal epilepsy compared to combined types of epilepsy supports the differences in treatment outcomes based on epilepsy subtype [30, 31]. Mechanistically, brivaracetam's unique pharmacological profile as a high-affinity ligand for SV2 A may confer differential efficacy in modulating neuronal excitability and neurotransmitter release in distinct epileptic foci [32]. Additionally, the adjunctive use of brivaracetam alongside existing ASMs may synergistically enhance seizure control through complementary mechanisms of action, further highlighting the importance of individualized treatment approaches in pediatric epilepsy management [33].

The long-term prognosis of childhood epilepsy hinges on factors such as remission, recurrence, seizure freedom, neurological impairment, and medication. Numerous clinical trials have reported the effective response rate of brivaracetam in treating epilepsy in adults. For instance, a phase IIb study reported responder rates for adjunctive brivaracetam treatment ranging from 30.8% to 39.6% [34]. In a phase III study, the responder rates for the 100 mg/day and the 200 mg/day brivaracetam groups were 38.9% and 37.8%, respectively [35]. The responder rate reported in our meta-analysis is higher than that reported in adult trials and a previous meta-analysis on pediatric epilepsy [36].

However, brivaracetam is not without adverse effects. The incidence of trial withdrawal due to TEAEs and poor efficacy was 10% and 14%, respectively. Furthermore, mental/behavior disorders and somnolence were reported in 6.8% and 8.09% of patients, respectively. Serious TEAEs affected 22.7% of patients, and severe TEAEs were noted in 11.8% of patients. Thus, although the efficacy outcomes are promising, the safety outcomes must be taken into consideration when managing patients with pediatric epilepsy.

Children undergoing treatment with newer ASMs experience a range of adverse reactions, including lethargy, accidental injury, vomiting, and behavioral issues like aggression, hostility, and hyperactivity. These adverse reactions are higher in children than adults [37]. For instance, Valproate's hepatotoxicity is particularly pronounced in children. In addition, Valproate is associated with more adverse behavioral effects compared to other ASMs, except levetiracetam [38, 39]. There are also common side effects like vomiting, drowsiness, and dizziness, especially seen with oxcarbazepine treatment [40]. Therefore, the safety profile of brivaracetam warrants careful consideration in clinical practice. The observed incidence of adverse events, including mental/behavior disorders and somnolence, underscores the need for vigilant monitoring and personalized risk–benefit assessment when prescribing brivaracetam to pediatric patients with epilepsy.

The retention rate of 66% indicates that the majority of patients continued the treatment despite these adverse effects, suggesting that the benefits of brivaracetam in controlling seizures may outweigh its potential risks. This retention rate was less than reported in the previous meta-analysis: 78% [36]. It was also lower than the 12-month retention rate observed in Toledo et al.'s study on adults [41]. Three primary factors contributed to the discontinuation: adverse events, poor efficacy, and patient preference. Across the included studies, TEAEs and poor efficacy were responsible for most treatment discontinuations. This highlights the importance of balancing therapeutic efficacy with tolerability and patient safety.

Our meta-analysis incorporates findings from a comprehensive array of studies, shedding light on the safety and efficacy of brivaracetam in pediatric epilepsy. Across the included studies, brivaracetam consistently emerged as a promising adjunctive therapy for refractory epilepsy in children.

Willems et al. [26] and Visa-Reñé et al. [25] highlighted brivaracetam's effectiveness in reducing seizure frequency, with a considerable proportion of patients experiencing seizure freedom. Although adverse effects were observed, they were generally manageable, indicating this population's favorable safety profile of brivaracetam.

Schubert-Bast et al. [24] and Russo et al. [19] offered further evidence supporting brivaracetam's efficacy and tolerability, mainly in focal epilepsy and epileptic encephalopathies. In these studies, brivaracetam demonstrated high retention rates and favorable responder rates, with few patients experiencing TEAEs.

Caraballo et al. [20] provided insights into BRV's effectiveness in challenging cases, such as developmental epileptic encephalopathies and refractory epilepsy. Despite the complexity of these conditions, brivaracetam still demonstrated significant seizure reduction and a favorable safety profile, suggesting its potential as a viable treatment option in difficult-to-manage cases.

McGuire et al. [23] and Liu et al. [18] contributed valuable data across different age groups. Furthermore, Hirsch et al. [22], Ferragut Ferretjans et al. [21], Farkas et al. [17], Lagae et al. [16], and Russo et al. [19] featured variations in study design and patient demographics. Despite that, their collective evidence still supports brivaracetam's efficacy and tolerability.

While individual studies may offer nuanced insights on brivaracetam in pediatric epilepsy, our meta-analysis contributes to a comprehensive understanding of its therapeutic profile. Despite its strengths, this meta-analysis has several limitations. Firstly, the possibility of publication bias cannot be entirely discounted, as studies with null or negative results may be underrepresented. Secondly, significant heterogeneity across studies may have influenced the pooled results, although sensitivity analysis was conducted to address this issue. Additionally, the generalizability of the findings may be limited to specific pediatric epilepsy populations or settings.

Furthermore, the relatively short duration of follow-up in some studies may restrict the assessment of long-term safety and efficacy outcomes. The absence of a comparator and the heterogeneous etiologies of epilepsy included may also limit the findings. In addition, the variation in doses makes it challenging to accurately interpret the findings. Lastly, potential confounding factors in observational studies could have influenced the observed outcomes despite attempts to control these factors through statistical adjustment or stratification.

The findings of this meta-analysis hold several implications for the field of pediatric epilepsy management. Firstly, the efficacy and tolerability of brivaracetam as adjunctive therapy in pediatric patients underscore its potential as a valuable treatment option. Clinicians may consider brivaracetam in their therapeutic armamentarium, especially for patients who have not responded adequately to conventional ASMs. Secondly, the observed safety profile of brivaracetam suggests its favourable risk–benefit profile in pediatric populations. This may provide reassurance to healthcare providers and patients regarding the use of brivaracetam in clinical practice. Thirdly, there was no standardized dosing of brivaracetam across the included studies. Lastly, our analysis has limitations: (1) a single-arm meta-analysis due to a lack of active comparators across the included studies, (2) the impossibility of performing a subgroup analysis based on electroclinical phenotype, epilepsy etiology, or the dose of brivaracetam.

Although we were limited by the data available, future research could explore potential prognostic factors associated with treatment response, such as age at epilepsy onset and seizure frequency. Additionally, the observed heterogeneity across the studies underscores the importance of standardizing study methodologies and outcome measures to facilitate more robust comparisons and meta-analyses. For instance, more studies utilizing brivaracetam at standardized doses are needed to strengthen the interpretation of pooled analyses. Furthermore, future studies can also explore varying doses of brivaracetam to provide insight into optimal dosing in pediatric epilepsy. Lastly, the need for long-term, prospective studies and randomized controlled trials evaluating the safety and efficacy of brivaracetam in diverse pediatric epilepsy populations remains paramount. Such studies could provide valuable insights into the sustained effects of brivaracetam treatment.

In conclusion, this meta-analysis highlights the efficacy, tolerability, and safety of brivaracetam as an adjunctive therapy in pediatric patients with epilepsy. The findings support its consideration as a valuable treatment option for children and adolescents, particularly those with drug-resistant epilepsy. However, further research is warranted to elucidate optimal dosing strategies, identify prognostic factors for treatment response, and assess long-term outcomes. Standardization of outcome measures and study methodologies would enhance comparability across studies and strengthen future meta-analyses. Overall, brivaracetam shows promise as an effective and well-tolerated therapeutic option for pediatric epilepsy, but ongoing research is needed to optimize its use in clinical practice.

## Supplementary Information

Below is the link to the electronic supplementary material.

Supplementary file1 (DOCX 194 KB)

## Data Availability

All data generated or analyzed during this study are included in this published article.
