# Supplementary material for: Efficacy and safety of Brivaracetam as adjunctive therapy in pediatric epilepsy: A systematic review and meta-analysis
Source: Neurol Sci. 2025 May 3;46(8):3525–36. doi: 10.1007/s10072-025-08185-9 (PMC12267362; doi:10.1007/s10072-025-08185-9)
Supplement: Supplementary file 1 — Supplementary file1 (DOCX 194 KB) [file 10072_2025_8185_MOESM1_ESM.docx]

**Risk of Bias Assessment Tables**

**Table 1S. Shows the quality assessment of the non-randomized clinical trials using ROBIN-1 tool**

| **Study** | **Confounding Bias** | **Selection Bias** | **Bias due to Classification of intervention** | **Deviation from intended interventions** | **Missing Data** | **Measurements of outcome Bias** | **Selection of Reported Results Bias** | **Overall Risk of Bias** |
| --- | --- | --- | --- | --- | --- | --- | --- | --- |
| Lagae 2023 | Low Risk | High Risk | Low Risk | Low Risk | High Risk | Low Risk | Low Risk | Low Risk |
| Farkas 2022 | Low Risk | Low Risk | Low Risk | Low Risk | Low Risk | Low Risk | Low Risk | Low Risk |
| Liu 2019 | Low Risk | Low Risk | Low Risk | Low Risk | Moderate Risk | Low Risk | Low Risk | Low Risk |

**Table 2S. Quality assessment of the observational cohort studies according to the Newcastle Ottawa Scale (NOS).**

| **Study** | **Selection** | | | | **Comparability** | **Outcome** | | | **quality** |
| --- | --- | --- | --- | --- | --- | --- | --- | --- | --- |
|  | **Representativeness of the exposed cohort** | **Selection of the non-exposed cohort** | **Ascertainment of exposure** | **the outcome of interest was not present at the start of the study** |  | **Assessment of outcome** | **Was follow-up long enough for outcomes to occur** | **Adequacy of follow-up of cohorts** |  |
| Caraballo 2024 | ⭐ | 0 | ⭐ | ⭐ | 0 | ⭐ | ⭐ | ⭐ | Good quality |
| Ferretjans 2021 | ⭐ | ⭐ | ⭐ | ⭐ | ⭐ | ⭐ | 0 | ⭐ | Good quality |
| Hirsch 2018 | ⭐ | ⭐ | ⭐ | 0 | 0 | ⭐ | ⭐ | ⭐ | Good quality |
| McGuire 2020 | ⭐ | ⭐ | ⭐ | 0 | 0 | ⭐ | ⭐ | ⭐ | Good quality |
| Russo 2022 | ⭐ | ⭐ | ⭐ | ⭐ | 0 | ⭐ | ⭐ | ⭐ | Good quality |
| Schubert-Bast 2018 | ⭐ | ⭐ | ⭐ | ⭐ | 0 | ⭐ | ⭐ | ⭐ | Good quality |
| Visa-Reñé 2020 | ⭐ | ⭐ | ⭐ | ⭐ | 0 | ⭐ | 0 | ⭐ | Good quality |
| Willems 2018 | ⭐ | ⭐ | ⭐ | ⭐ | 0 | ⭐ | ⭐ | ⭐ | Good quality |

**
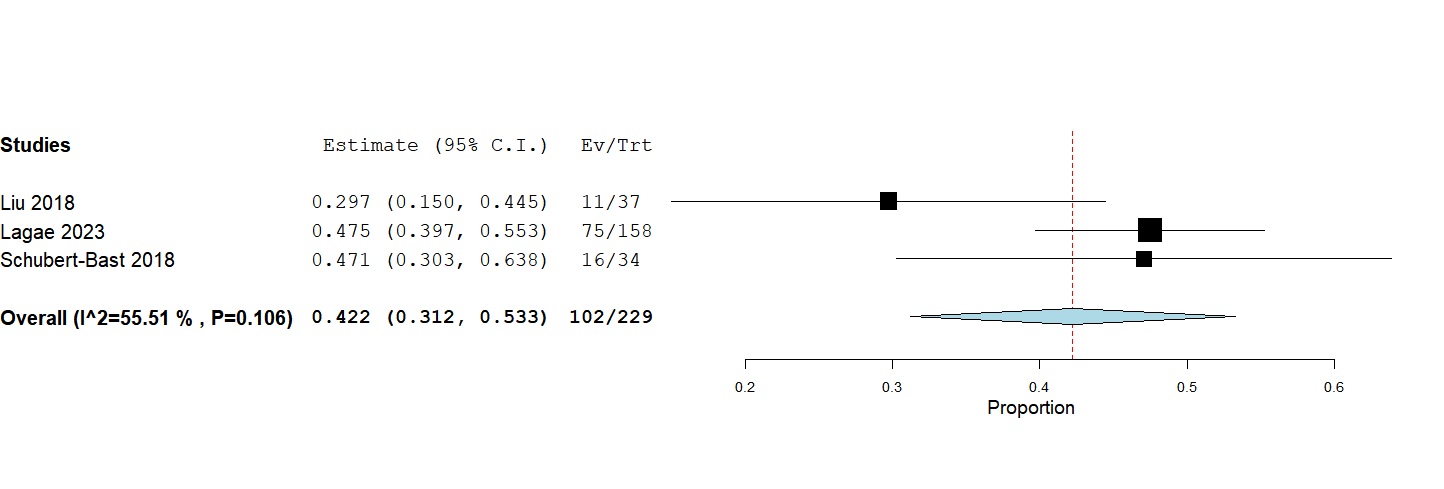
Figure 1S. Leave-one-out analysis of the more than 50% response rate in focal epilepsy**

**
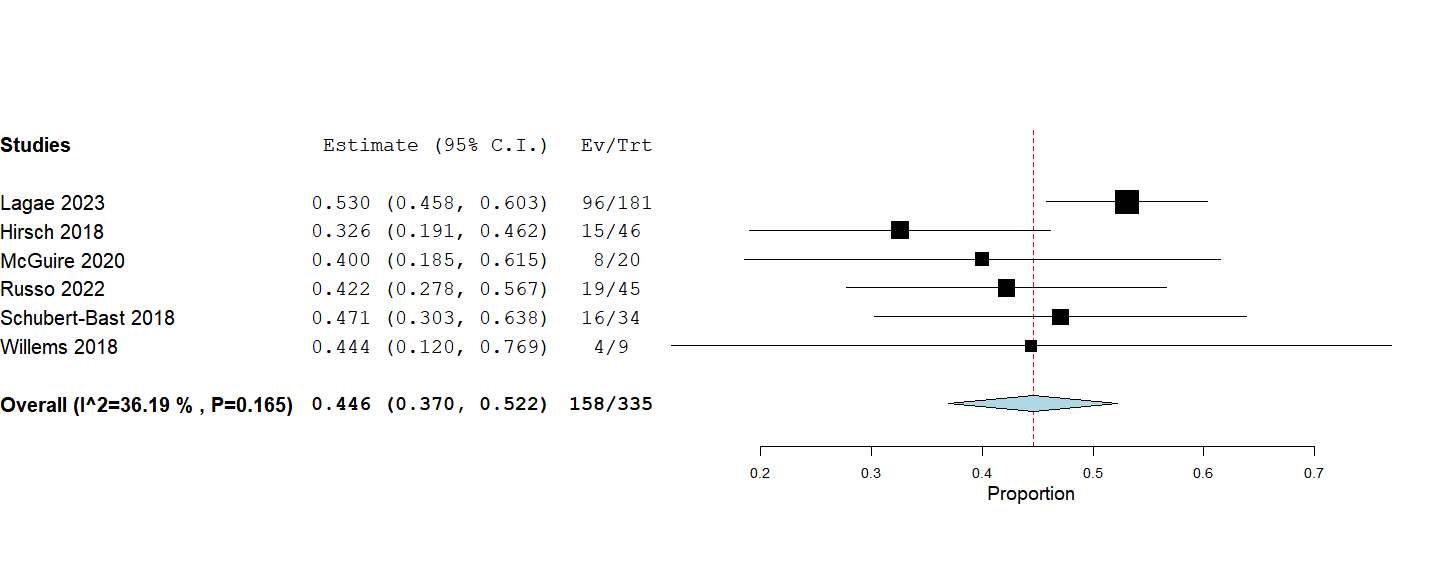
Figure 2S. Leave-one-out analysis of the more than 50% response rate in combined epilepsy types.**

**
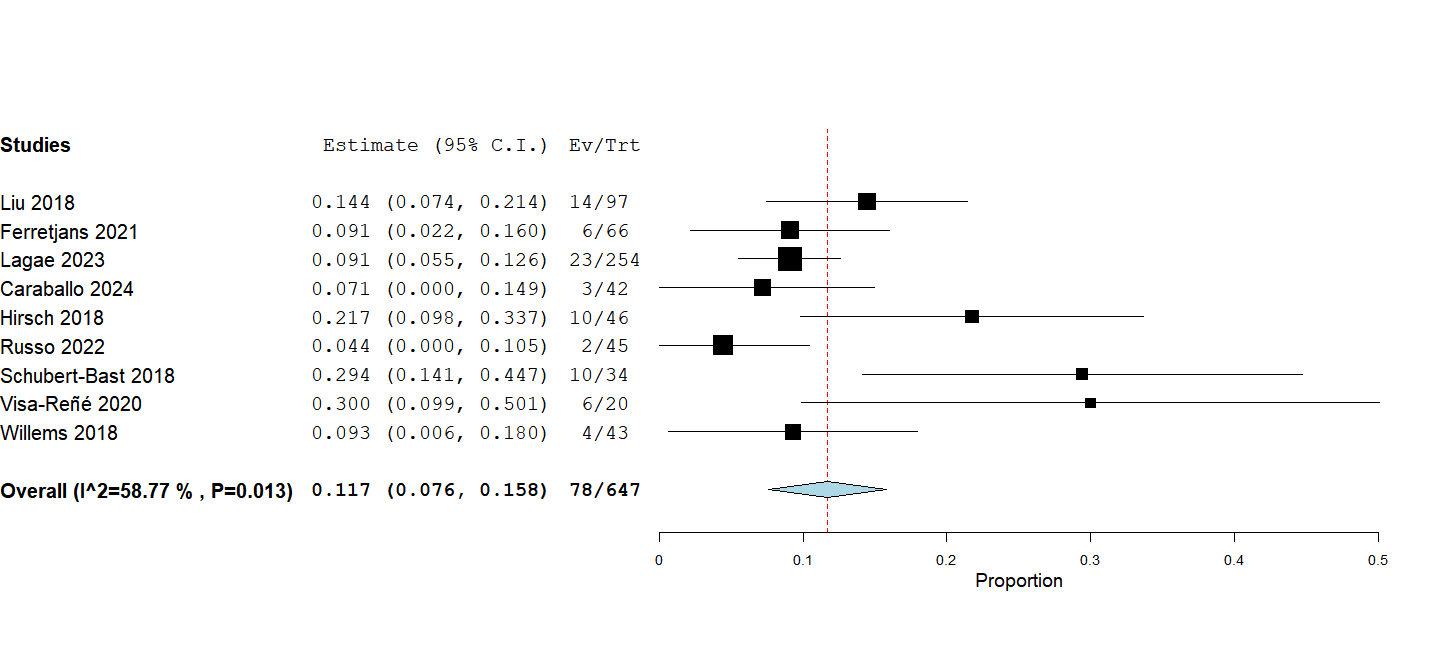
Figure 3S. Analysis of complete seizure freedom in combined epilepsy types.
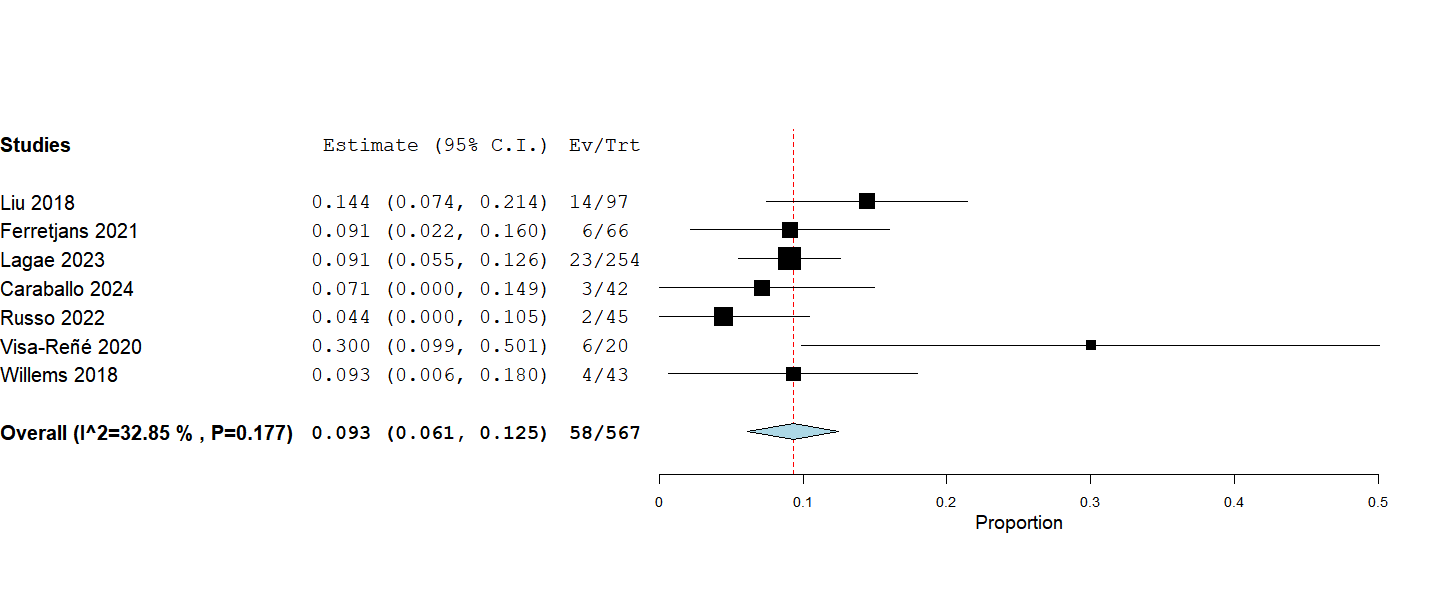
Figure 4S. Leave one out analysis of complete seizure freedom in combined epilepsy types**


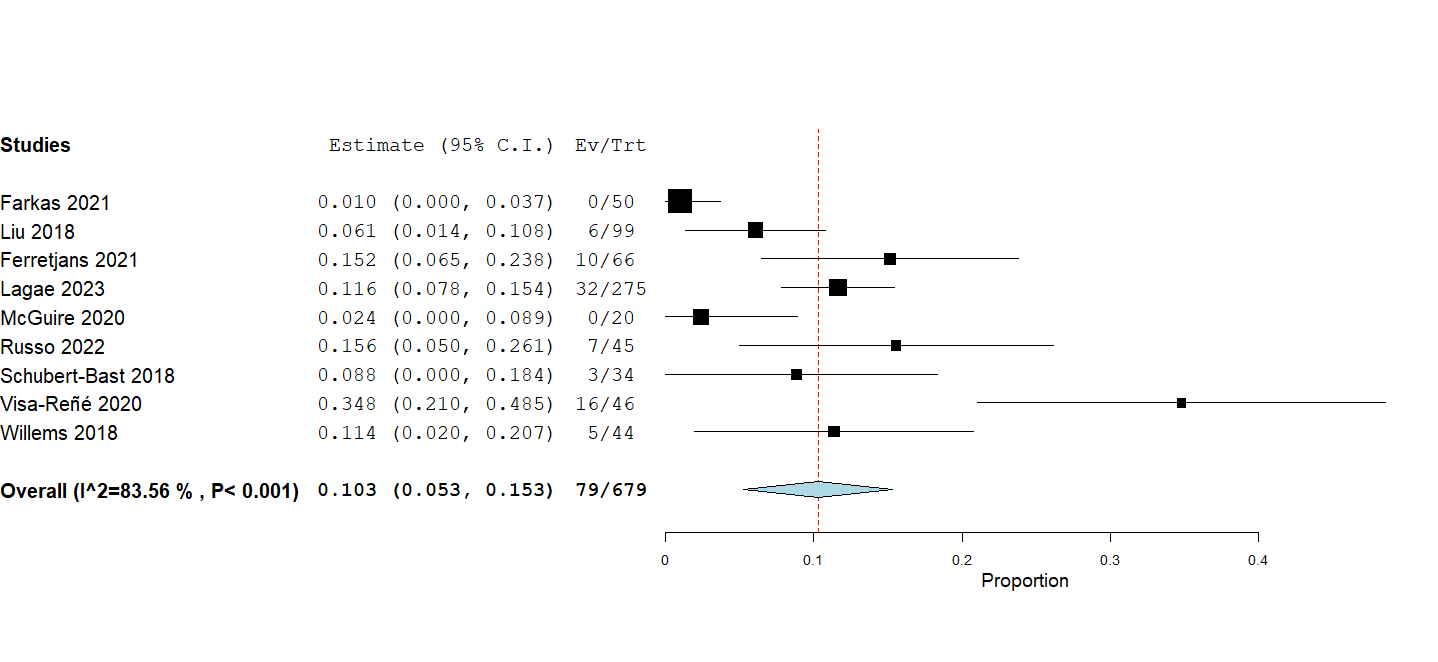


**Figure 5S. Analysis of trail withdrawal due to TEAEs**


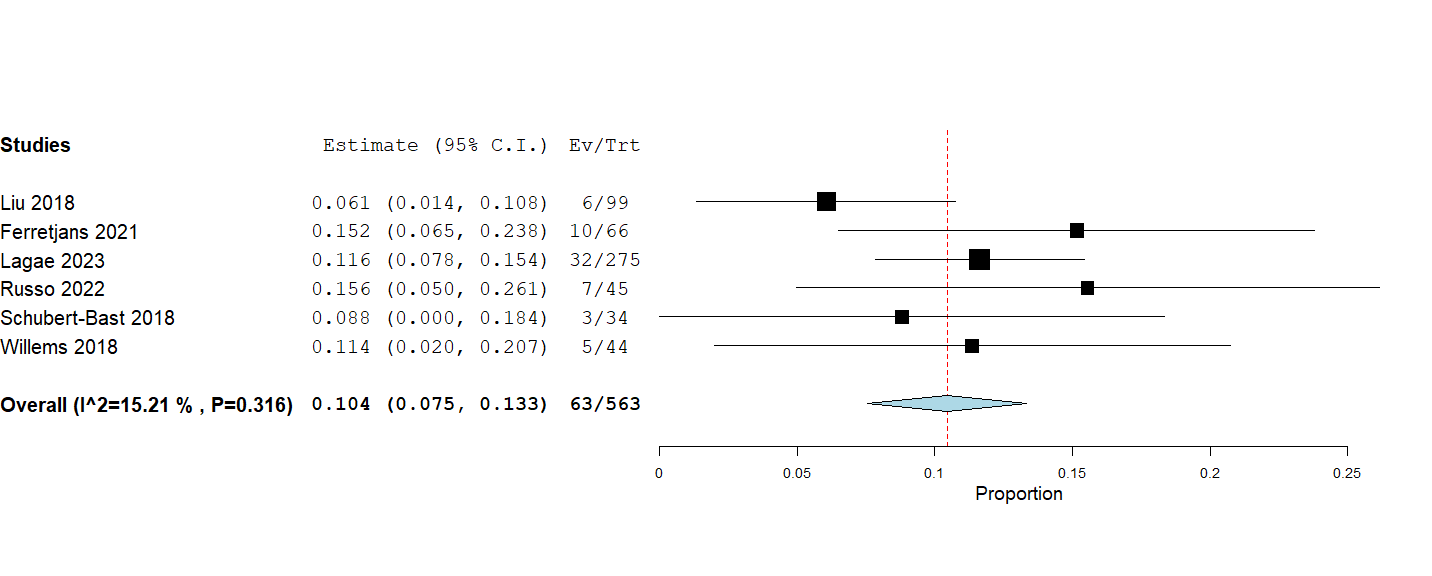


**Figure 6S. Leave one out analysis of trail withdrawal due to TEAEs**


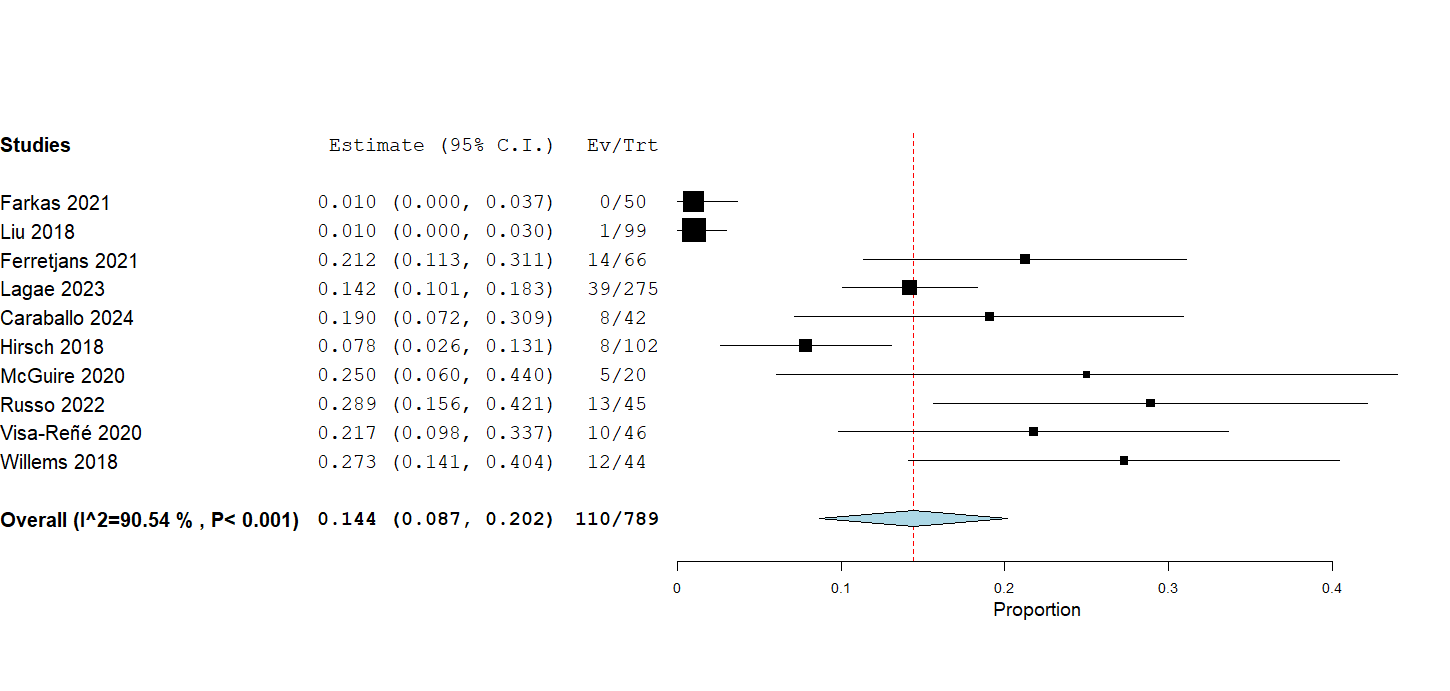


**Figure 7S. Analysis of trial withdrawal due to poor efficiency**


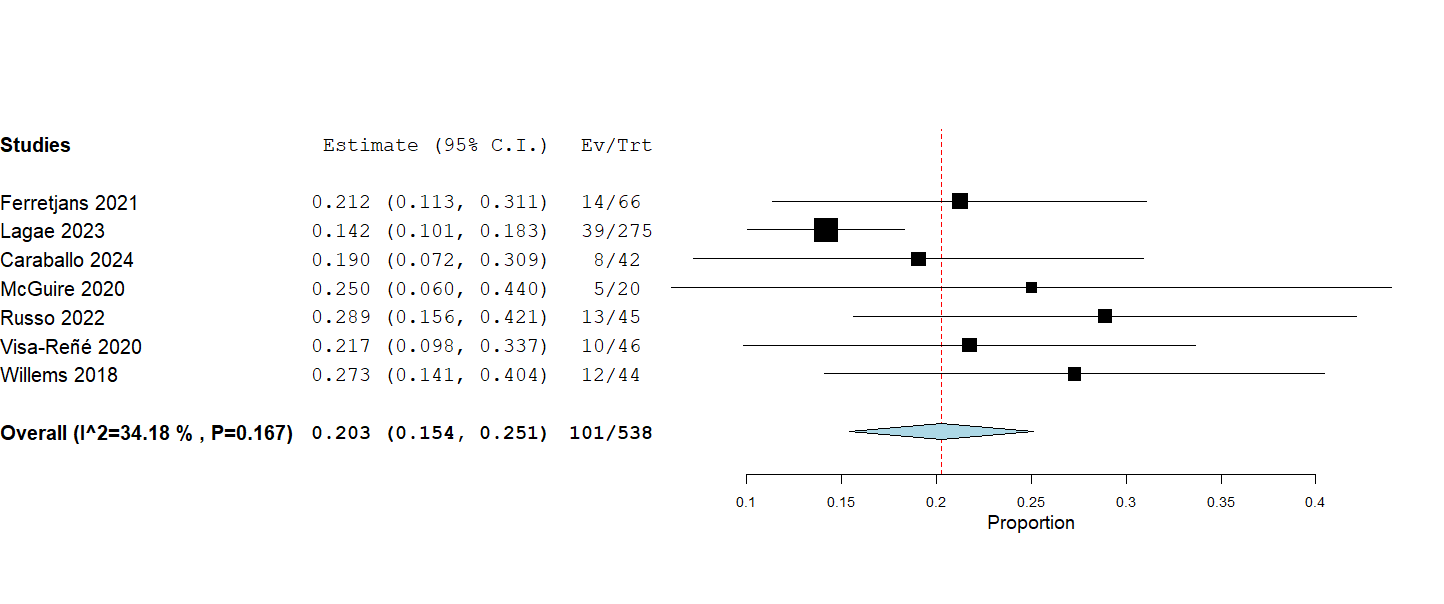


**Figure 8S. Leave one out analysis of trial withdrawal due to poor efficiency**


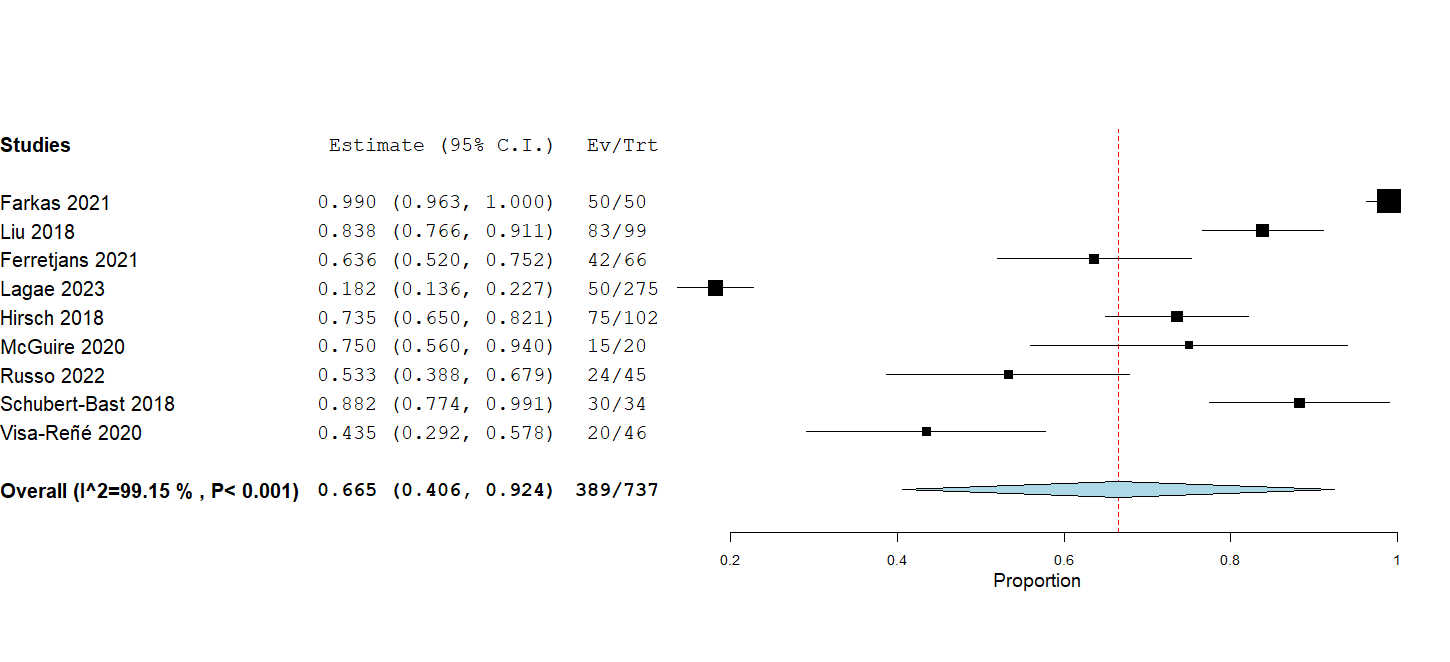


**Figure 9S. Analysis of retention rate**


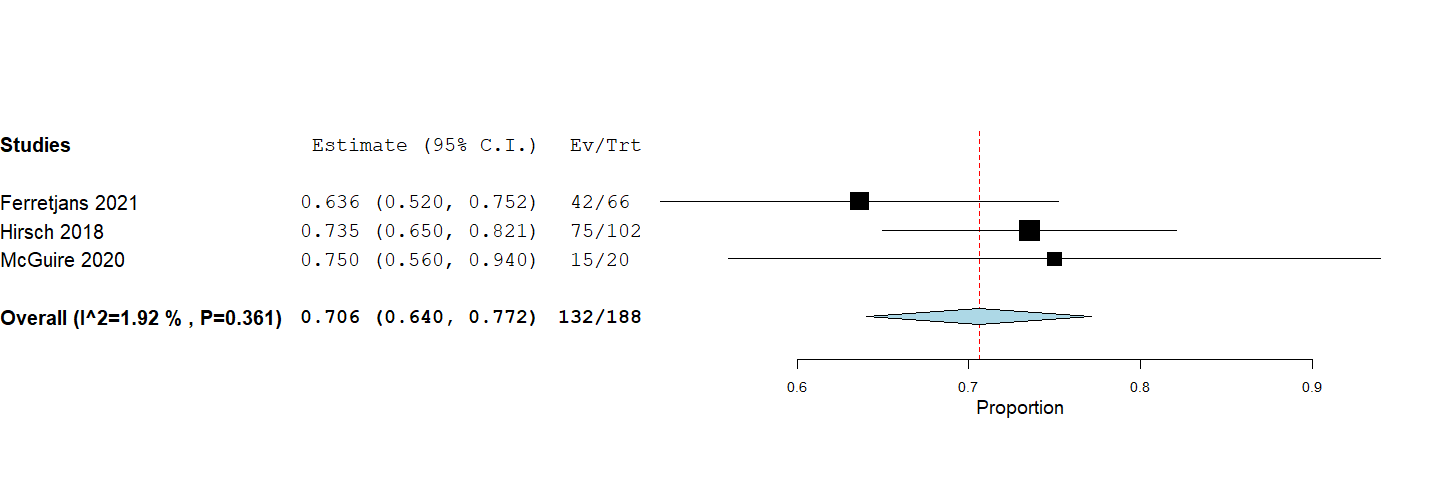


**Figure 10S. Leave one out analysis of the retention rate**
